# Urban–Rural Differences in Hip Fracture Mortality: A Nationwide NOREPOS Study

**DOI:** 10.1002/jbm4.10236

**Published:** 2019-10-21

**Authors:** Siri M Solbakken, Jeanette H Magnus, Haakon E Meyer, Cecilie Dahl, Hein Stigum, Anne J Søgaard, Kristin Holvik, Grethe S Tell, Nina Emaus, Siri Forsmo, Clara G Gjesdal, Berit Schei, Peter Vestergaard, Tone K Omsland

**Affiliations:** ^1^ Institute of Health and Society, Department of Community Medicine and Global Health University of Oslo Oslo Norway; ^2^ Faculty of Medicine University of Oslo Oslo Norway; ^3^ Department of Chronic Diseases and Ageing Norwegian Institute of Public Health Oslo Norway; ^4^ Department of Global Public Health and Primary Care University of Bergen Bergen Norway; ^5^ Department of Health and Care Sciences The Arctic University of Norway Tromsø Norway; ^6^ Department of Public Health and Nursing Norwegian University of Science and Technology Trondheim Norway; ^7^ Department of Clinical Science University of Bergen Bergen Norway; ^8^ Department of Rheumatology Haukeland University Hospital Bergen Norway; ^9^ Department of Gynecology St Olavs Hospital Trondheim Norway; ^10^ Department of Clinical Medicine Aalborg University Aalborg Denmark; ^11^ Department of Endocrinology Aalborg University Hospital Aalborg Denmark; ^12^ Steno Diabetes Center North Jutland Aalborg Denmark

**Keywords:** AGING, EPIDEMIOLOGY, GENERAL POPULATION STUDIES, OSTEOPOROSIS, STATISTICAL METHODS

## Abstract

Higher hip fracture incidence in urban than in rural areas has been demonstrated, but urban–rural differences in posthip fracture mortality have been less investigated, and the results are disparate. Hence, the aims of the present register‐based cohort study were to examine possible urban–rural differences in short‐ and long‐term mortality in Norwegian hip fracture patients and their potential associations with sociodemographic variables, and to investigate possible urban–rural differences in excess mortality in hip fracture patients compared with the general population. Data were provided from the NOREPOS hip fracture database, the 2001 Population and Housing Census, and the National Registry. The urbanization degree in each municipality was determined by the proportion of inhabitants living in densely populated areas (rural: <1/3, semirural: 1/3 to 2/3, and urban: >2/3). Age‐adjusted mortality rates and standardized mortality ratios were calculated for hip fracture patients living in rural, semirural, and urban municipalities. A flexible parametric model was used to estimate age‐adjusted average and time‐varying HRs by category of urbanization with the rural category as reference. Among 96,693 hip fracture patients, urban residents had higher mortality than their rural‐dwelling counterparts. The HR of mortality in urban compared with rural areas peaked during the first 1 to 2 years postfracture with a maximum HR of 1.20 (95% CI, 1.10 to 1.30) in men and 1.15 (95% CI, 1.08 to 1.21) in women. The differences were significant during approximately 5 years after fracture. Adjusting for sociodemographic variables did not substantially change the results. However, absolute 30‐day mortality was not significantly different between urban and rural residents, suggesting that health‐care quality immediately postfracture does not vary by urbanization. The novel findings of a higher long‐term mortality in urban hip fracture patients might reflect disparities in health status or lifestyle, differences in posthip fracture health care or rehabilitation, or a combination of several factors. © 2019 The Authors. *JBMR Plus* published by Wiley Periodicals, Inc. on behalf of American Society for Bone and Mineral Research.

## Introduction

Hip fractures represent a major health problem in Western societies, and numbers are likely to increase with an aging population.^1^ Hip fractures are associated with considerably impaired function and reduced quality of life in the individual patient, and constitute a major economic burden on the health‐care systems.^2^, ^3^, ^4^, ^5^ There is also a considerably increased posthip fracture mortality,^6^ which is associated with numerous risk factors, both in terms of individual patient characteristics such as comorbidity, age, and sex, as well as health‐care system‐related factors, such as waiting time to surgery.^7^, ^8^ Urbanization is interesting when addressing posthip fracture mortality, as transportation time to hospital and the quality of health‐care services may differ between urban and rural areas, and this could possibly affect mortality. Furthermore, differences in health status and sociodemographic factors, such as educational level and social network, could potentially contribute to urban–rural mortality differences following hip fractures.

Whereas urban–rural differences in hip fracture incidence are well‐documented with higher rates in urban areas,^9^ urban–rural differences in posthip fracture mortality have been less studied. The results from previous studies are somewhat disparate,^10^, ^11^, ^12^ and we identified only one small study on urban–rural differences in long‐term (5 years) mortality.^11^ Furthermore, if mortality in the general population differs by category of urbanization, this could potentially explain any observed urban–rural differences in hip fracture mortality. None of the previous studies report measures of excess mortality by comparing mortality in hip fracture patients to the general population mortality in urban and rural areas. Thus, possible underlying urban–rural mortality differences in the general population have not been taken into consideration. Therefore, the aims of the current study were to examine: (1) any urban–rural differences in short‐term and long‐term absolute mortality; (2) any urban–rural differences in short‐term and long‐term relative mortality; (3) whether possible urban–rural differences could be explained by sociodemographic factors such as level of education, number of children, and whether the patient lived alone or not; and (4) urban–rural differences in excess mortality in hip fracture patients compared with the general population.

## Materials and Methods

### The NOREPOS hip fracture database (NORHip)

The study was based on hip fractures sustained during 2002 to 2013 in patients aged 50 to 100 years. Data from all Norwegian hospitals treating hip fractures during 1994 to 2013 are available from the nationwide NOREPOS hip fracture database (NORHip).^13^ As only first hip fractures were included in the study, the period 1994 to 2001 (before the Population and Housing Census, see below) was used as a washout period. Detailed descriptions of the data collection, classification, validation, and quality assurance of the NORHip are available^14^, ^15^ and are briefly outlined below. For the period 2002 to 2007, the identification of hip fractures was based on computerized discharge diagnoses (ICD‐10 S72.0‐S72.2) from the patient administration systems in the hospitals. Additionally, dates of admission and discharge, additional diagnosis codes, and surgical procedure codes registered during the hip fracture hospitalization were also retrieved.^14^ For the period 2008 to 2013, NORHip was updated with data from the Norwegian Patient Registry, using the same procedure when identifying fractures as in the first NORHip data collection.^15^ Both NORHip data collections have been thoroughly validated and quality assured.^14^, ^15^ Subjects entered the cohort on the day of their first hip fracture and were followed until death (event), emigration, or end of follow‐up on October 31, 2014. Dates of death and emigration in hip fracture patients and in the general population were retrieved from the National Registry.

### Definition of rural, semirural, and urban municipalities

The 2001 Population and Housing Census (Statistics Norway) was used to obtain information on municipality of residence for hip fracture patients and the general Norwegian population. According to Statistics Norway, geographic entities for which inhabitants are estimated to live less than 50 meters apart, are defined as being densely populated. The number of individuals living in densely populated areas within different municipalities is available online.^16^ For each municipality a degree of urbanization was calculated as a continuous variable (between 0 and 1), reflecting the proportion of inhabitants living in densely populated areas in 2001. In municipalities with an urbanization degree of 0, no one lived in densely populated areas, whereas 1 corresponded to everyone living in densely populated areas. The municipalities were categorized in three groups according to the proportion living in densely populated areas: rural (<1/3), semirural (1/3 to 2/3), and urban (>2/3).

### Sociodemographic factors

Information about the highest achieved level of education, number of children, and whether the patient lived alone was retrieved from the 2001 Population and Housing Census. Three categories of educational level were defined: primary education (0 to 9 years of education), secondary education (10 to 12 years of education), and tertiary education (≥13 years of education). Information on one or more of these variables was missing for 3177 hip fracture patients (3.3%). As the proportion of missing data was small, these patients were excluded from the multivariable adjusted analyses.

### Statistics

#### *Analyses of absolute mortality in hip fracture patients*

Data were analyzed in Stata SE 15 (Stata Corp., College Station, TX, USA). Age‐adjusted mortality rates were calculated by sex and urbanization category for defined time intervals (0 to 30 days, 1 to 12 months, >1 to 5 years, and 5.1 to 12.8 years after the fracture) by direct standardization.

#### *Analyses of relative mortality in hip fracture patients*

In model‐based analyses of survival, a flexible parametric survival model was used as an alternative to the Cox model to allow nonproportional hazards and predict time‐varying HRs.^17^ We compared model fit with and without urbanization category (exposure) as a time‐varying coefficient (based on likelihood ratio, and the Akaike and the Bayesian information criterion). When used as a time‐varying coefficient, the effect of urbanization on mortality is assumed to vary over follow‐up time.^17^ Separate models were fitted for men and women. Age‐adjusted HRs by category of urbanization were first calculated for the whole time period with the rural category as reference. These analyses, which reported an average HR for the whole period of follow‐up, were run without a time‐varying coefficient. In analyses used to predict the HR for hip fracture patients in urban compared with rural areas by time after fracture, urbanization category was used as a time‐varying coefficient to detect a possible variation in HR during the follow‐up time caused by time‐varying effects. Furthermore, statistical interactions were tested between urbanization category and educational level, number of children, county, and the variable indicating whether a patient lived alone.

#### *Analyses of excess mortality in hip fracture patients compared with the general population*

In analyses of excess mortality in hip fracture patients, sex‐specific mortality in 0.5‐year‐age groups by calendar year in the Norwegian population (2002 to 2014) was used as reference. As we did not have information on birth date and month (only year), July 1 was used as an approximate birth date for all patients when calculating person time; therefore, 0.5‐year‐ age groups had to be used to categorize subjects correctly by age group and calendar year. Age‐adjusted mortality rates in the general population by urbanization category and sex were calculated. Excess mortality was first estimated by calculating standardized mortality ratios (SMRs), and then by using the flexible parametric survival model.

SMRs with 95% CIs were calculated by dividing age, sex, and calendar‐year‐specific mortality rates in hip fracture patients by the corresponding mortality rates in the general population. Total and 1‐year SMRs were first calculated for male and female hip fracture patients, regardless of urbanization category, to obtain an overall estimate of excess mortality in hip fracture patients comparable to previous studies. Second, SMRs for the whole period were calculated within each urbanization category. In the latter case, the mortality rates used as reference were also specific for each category of urbanization (in addition to being age‐, sex‐, and calendar‐year‐specific).

Finally, the flexible parametric survival model was also used to investigate potential differences in excess mortality across degree of urbanization. In this model, sex‐specific excess mortality rate ratios were estimated by comparing excess hazard rates in urban, semirural, and rural areas. Again, the general population mortality by 0.5‐year age group, sex, calendar year, and urbanization category was used as reference.

#### *Sensitivity analyses*

The potentially long timespan between the Population and Housing Census and the hip fracture could result in misclassification if the patient had changed place of residence during this period. Therefore, sensitivity analyses excluding patients who fractured after 2006 (ie, more than 5 years after the Population and Housing Census) were performed. Moreover, urbanization degree was determined on municipality level. If urbanization degree varied within a municipality, this might have led to misclassification of some inhabitants. Therefore, sensitivity analyses using urbanization degree as a continuous variable were also conducted to compare patients living in municipalities with an urbanization degree of 1 (strictly urban) to patients living in municipalities with an urbanization degree of 0 (strictly rural).

### Ethics

The study and the linkages of data were approved by the Norwegian Data Inspectorate, the Directorate of Health, Statistics Norway, The National Registry, the Norwegian Patient Registry, and the Regional Committee for Medical and Health Research Ethics.

## Results

### Characteristics of the study population

The study included 96,693 first‐time hip fracture patients aged 50 to 100 years (69.8% women). There were 62,317 hip fracture patients who died during the study period (64.4%). The majority of patients lived in urban areas (66.7% of men and 69.3% of women; Table 1), and mean age at hip fracture was slightly lower in urban than in rural municipalities (*p* < 0.001 for the difference in both sexes). In men, median survival time was 2.7 years in all categories of urbanization. The corresponding median survival times in women were 4.3, 4.3, and 4.1 years in rural, semirural, and urban areas, respectively. In both sexes, the proportion of hip fracture patients with primary education only (0 to 9 years) was highest in the rural municipalities.

**Table 1:** Descriptive Data of Norwegian Hip Fracture Patients Aged 50–100 Years by Urbanization Category and Sex. The NOREPOS Hip Fracture Database (2002 to 2013)

	Rural areas	Semirural areas	Urban areas	Total
Men				
Total number of patients with hip fracture	2364	7363	19,503	29,230
Total number of person years	7161	22,215	57,805	87,180
Total number of deaths (%)	1626 (68.8)	5038 (68.4)	13,354 (68.5)	20,018 (68.5)
Total number of deaths during the first year (%)	738 (31.2)	2385 (32.4)	6302 (32.3)	9425 (32.2)
Mean age at time of hip fracture in years (SD)^a^	79.8 (10.6)	79.2 (10.4)	78.4 (10.6)	78.7 (10.6)
Median survival time in years (interquartile range)	2.7 (6.6)	2.7 (6.8)	2.7 (6.7)	2.7 (6.7)
Number of patients with primary education only (%)^a^^,^ b ^,^ c	1303 (55.4)	3645 (50.0)	6994 (36.3)	11,942 (41.3)
Mean number of children per patient^b^	2	2	2	2
Number of patients living alone (%)^a^^,^ b	858 (37.1)	2348 (32.5)	6328 (33.0)	9534 (33.2)
Women				
Total number of patients with hip fracture	4952	15,755	46,756	67,463
Total number of person years	18,943	59,264	171,783	249,990
Total number of deaths (%)^a^	3158 (63.8)	9676 (61.4)	29,465 (63.0)	42,299 (62.7)
Total number of deaths during the first year (%)^a^	1013 (20.5)	3156 (20.0)	9994 (21.4)	14,163 (21.0)
Mean age at time of hip fracture in years (SD)^a^	82.3 (9.1)	81.7 (9.5)	81.6 (9.4)	81.7 (9.4)
Median survival time in years (interquartile range)	4.3 (7.0)	4.3 (7.5)	4.1 (7.2)	4.2 (7.3)
Number of patients with primary education only (%)^a^^,^ b ^,^ c	3091 (62.8)	9282 (59.3)	23,159 (50.0)	35,532 (53.2)
Mean number of children per patient^b^	2	2	2	2
Number of patients living alone (%)^a^^,^ b	2689 (56.0)	8169 (53.3)	26,490 (58.1)	37,348 (56.8)

a Statistically significant difference (*p* ≤ 0.001) between two or more categories of urbanization.

b A total of 3.3% of the patients had missing data on the variables education, number of children, and/or whether they were living alone.

c Primary education: Maximum 9 years of education.

### Urban–rural differences in absolute mortality

**Figure 1:**
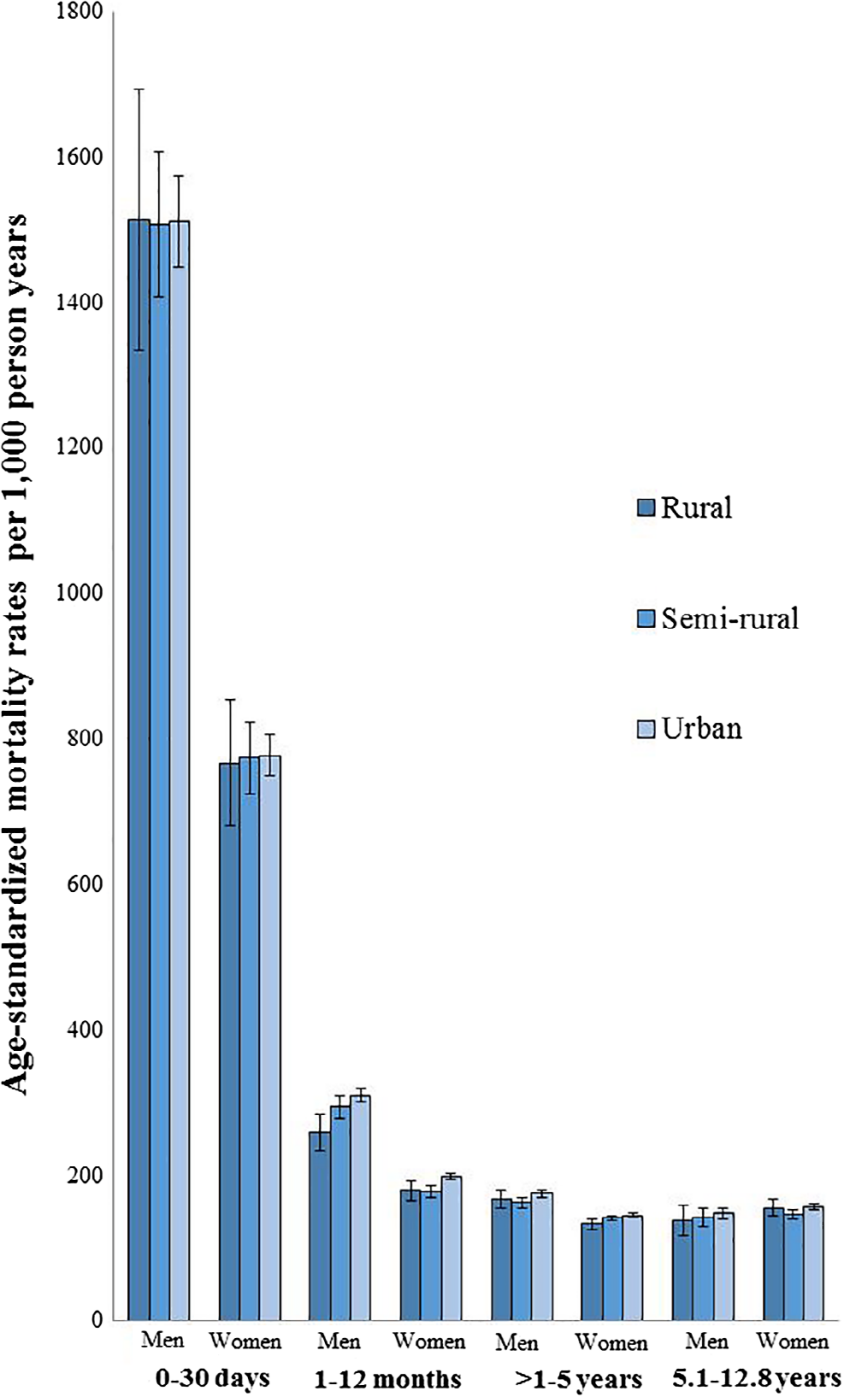
Age‐adjusted mortality rates with 95% CIs in hip fracture patients, by sex, urbanization category, and time after hip fracture. Norwegian hip fracture patients aged 50 to 100 years. The NOREPOS Hip Fracture Database (2002 to 2013).

Age‐adjusted 30‐day mortality rates were not statistically significantly different between urban, semirural, and rural residents (Fig. 1). After 30 days, increasing degree of urbanization was associated with higher mortality. During the period 1 to 12 months postfracture, a significant urban–rural difference in absolute mortality was demonstrated, with 19% higher rates in urban compared with rural areas among men [age‐adjusted mortality rates 310 (95% CI, 300 to 319) versus 260 (95% CI, 235 to 284) per 1000 person years], and 11% higher rates among women [age‐adjusted mortality rates 198 (95% CI, 193 to 203) versus 179 (95% CI, 166 to 192) per 1000 person years]. This urban–rural difference was also observed beyond the first year in women, although less pronounced [age‐adjusted mortality 145 (95% CI, 143 to 148) versus 133 (95% CI, 126 to 140) per 1000 person years during the period >1 to 5 years postfracture].

### Urban–rural differences in relative mortality and sociodemographic factors

**Figure 2:**
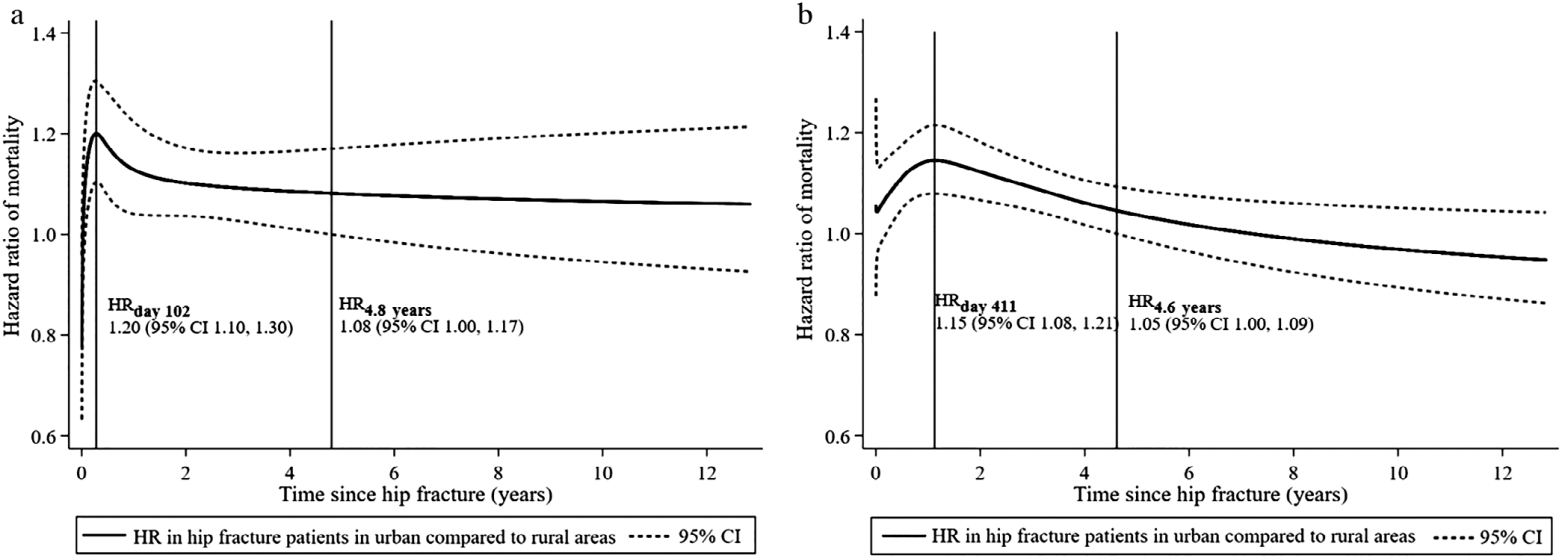
Predicted relative mortality in patients living in urban compared with rural municipalities by time after hip fracture in (*A*) men and (*B*) women. Two points in time during follow‐up and their corresponding HRs with 95% CIs are specified with reference lines: (1) when the HR reaches maximum, and (2) when the HR is no longer significant (when the lower confidence interval intersects the line corresponding to a HR of 1). Norwegian hip fracture patients aged 50 to 100 years. The NOREPOS Hip Fracture Database (2002 to 2013).

Overall, mortality was 8% higher in hip fracture patients living in urban compared with rural areas during the 12.8 years of follow‐up [average HR for the whole period 1.08 (95% CI, 1.05 to 1.11), adjusted for age and sex]. There were no significant differences between semirural and rural areas (HR 1.02; 95% CI, 0.99 to 1.05). Adjusting for educational level, county of residence, whether the patient lived alone, and number of children did not materially change the estimates. There were no significant interactions between these variables and urbanization category. In analyses where urbanization category was used as a time‐varying coefficient, the HR of mortality between rural and urban municipalities clearly varied by time after hip fracture. In men, a peak during the first year after hip fracture was seen, reaching a maximum HR of 1.20 (95% CI, 1.10 to 1.30) on day 102 postfracture (Fig. 2 *A*). The HR gradually declined thereafter, but remained significant until 4.8 years after the hip fracture. In women, the peak in HR appeared later [HR 1.15 (95% CI, 1.08 to 1.21) after 411 days], and was no longer significant after 4.6 years (Fig. 2 *B*).

### Excess mortality in hip fracture patients compared with the general population

When estimating the excess mortality in hip fracture patients using mortality in the Norwegian population (2002 to 2014) by sex and 0.5‐year‐age groups as reference, the overall SMRs relative to the expected sex‐, age‐, and calendar‐year‐specific mortality for the whole period were 2.14 (95% CI, 2.11 to 2.17) in male hip fracture patients and 1.41 (95% CI, 1.40 to 1.42) in female hip fracture patients. The corresponding SMRs for the first year after fracture were 4.90 (95% CI, 4.80 to 5.00) in men and 3.01 (95% CI, 2.96 to 3.06) in women, respectively.

### Urban–rural differences in excess mortality

**Figure 3:**
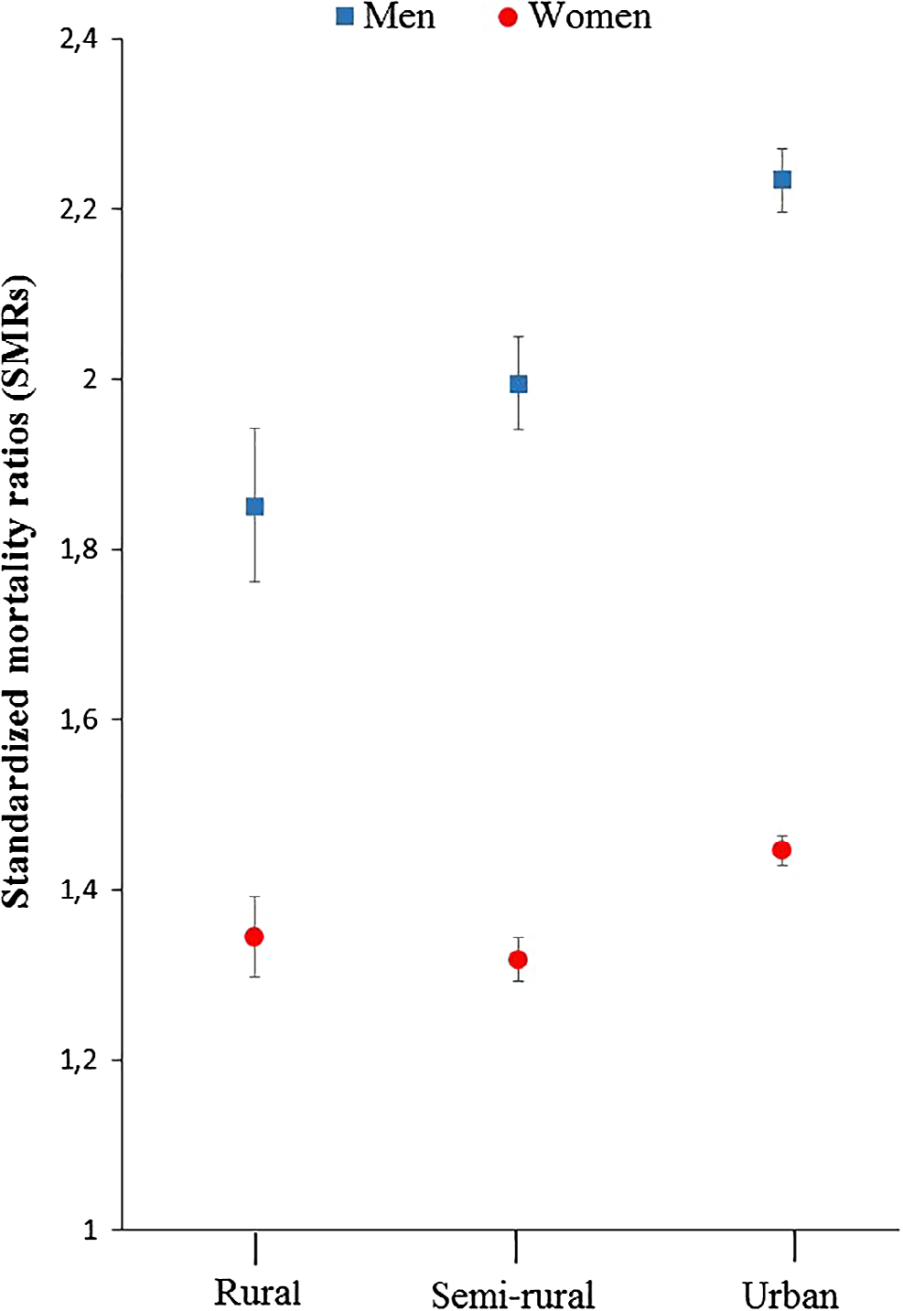
Standardized mortality ratios (SMRs) by category of urbanization with 95% CIs in hip fracture patients (men and women aged 50 to 100 years from the NOREPOS Hip Fracture Database, 2002 to 2013), relative to the expected sex, age, calendar year, and urbanization category‐specific mortality in the general population without hip fracture.

In the general population, absolute mortality was similar across categories of urbanization during the study period: age‐adjusted mortality rates in men 234 (95% CI, 230 to 237), 234 (95% CI, 230 to 234), and 230 (95% CI, 230 to 234) per 10,000 person years in rural, semirural, and urban areas, respectively. The corresponding mortality rates in women in the general population were 201 (95% CI, 197 to 204), 204 (95% CI, 204 to 208), and 204 (95% CI, 204 to 208) per 10,000 person years. In analyses of urban–rural differences in excess mortality, SMRs in male hip fracture patients for the whole period were 1.85 (95% CI, 1.76 to 1.94), 1.99 (95% CI, 1.94 to 2.05), and 2.23 (95% CI, 2.20 to 2.27) in rural, semirural, and urban areas, respectively. As for male hip fracture patients, the highest excess mortality in female hip fracture patients was found in urban areas: SMR 1.45 (95% CI, 1.43 to 1.46). The corresponding SMRs for female hip fracture patients in semirural and rural areas were 1.32 (95% CI, 1.29 to 1.34) and 1.34 (95% CI, 1.30 to 1.39), respectively (Fig. 3).

Excess mortality estimated by the flexible parametric survival model demonstrated similar and significant relative differences in excess mortality between urban and rural areas in both men and women. In this model, there were no significant differences in excess mortality between semirural and rural areas.

### Sensitivity analyses

Sensitivity analyses, excluding patients with more than 5 years between the Population and Housing Census in 2001 and the hip fracture, demonstrated the same patterns as the main analyses (results not shown). Furthermore, analyses comparing municipalities with urbanization degree 1 versus 0 (ie, strictly urban municipalities where all inhabitants lived in urban areas versus strictly rural municipalities where all inhabitants lived in rural areas) yielded similar results as the main analyses (results not shown).

## Discussion

In this study of more than 90,000 first‐time hip fracture patients, increasing degree of urbanization was associated with a higher posthip fracture mortality when considering the total follow‐up time of 12.8 years. This urban–rural difference in mortality was present in both men and women, varied over time, peaked during the first 1 to 2 years after fracture, and remained statistically significant until approximately 5 years postfracture. Furthermore, the highest excess mortality was found among hip fracture patients living in urban municipalities. However, absolute mortality the first 30 days after hip fracture did not differ significantly between urban and rural municipalities, indicating that the quality of the acute hospital care offered to this patient group does not vary by urbanization.

Previous studies have demonstrated urban–rural differences in hip fracture incidence, as summarized in a systematic review,^9^ but variation in hip fracture mortality by degree of urbanization has been less studied. Miller and colleagues^10^ found increased in‐hospital mortality, but a lower 1‐year mortality among rural compared with urban hip fracture patients in the United States. However, urban–rural differences were small despite a greater travel distance to treatment facility among patients residing in rural areas. In contrast, Weller and colleagues^12^ reported a nonsignificant trend towards decreased mortality among hip fracture patients treated in urban versus rural community hospitals, both in‐hospital and after 3, 6, and 12 months. We could only identify one small study that investigated urban–rural mortality differences beyond the first year postfracture. In contrast to our results, they did not report any significant differences at 1 or 5 years after fracture.^11^ The above‐mentioned studies have either defined urban and rural municipalities in different ways^10^, ^12^ or the definitions are unclear,^11^ thus direct comparisons with our results should be interpreted with some caution. Keeping this in mind, our study partly diverges from previous findings and is, to the best of our knowledge, the first to demonstrate urban–rural differences in hip fracture mortality up to 5 years postfracture, and to identify a distinct peak in the HR between urban and rural areas during the first 1 to 2 years.

In Norwegian municipalities, travel time to the nearest hospital with an emergency department ranges from a few minutes to up to more than 4 hours.^18^ However, no significant urban–rural differences in absolute 30‐day mortality were observed, and this finding suggests that distance to hospital does not play an important role for survival in this setting and that hip fracture patients in urban and rural municipalities generally have access to similar health‐care quality immediately after the fracture. In line with this, 30‐day survival after hip fracture varies only slightly between hospital trusts in Norway, although a report on this quality indicator did not distinguish between patients from rural and urban municipalities.^19^ However, it should be kept in mind that other indicators of health‐care quality in hip fracture patients might be highly relevant, substantiated by the fact that the most distinct urban–rural mortality differences were observed during the first 1 to 2 years after hip fracture in our study. One possible explanation for this interesting pattern could be inequalities between urban and rural municipalities regarding follow‐up health‐care or rehabilitation services. Additional studies using different indicators are therefore needed to determine whether follow‐up health‐care services differ by geographic location.

Several other factors might contribute to explain the urban–rural mortality differences observed during the first years after hip fracture. Differences in general health status and comorbidity level between urban and rural areas could be an important reason. A study of more than 11,000 women in Norway demonstrated lower BMD, lower BMI, and a higher proportion with poor or fair self‐reported health among urban compared with rural women,^20^ indicating a higher level of frailty among urban residents. These findings will likely apply to our hip fracture patients as well, and could potentially explain, at least in part, the increased mortality among urban patients. On the other hand, a higher burden of comorbidity among hip fracture patients in urban areas could also be expected to result in higher 30‐day mortality in these patients; however, this was not the case in our study.

Although sociodemographic indicators including education might influence mortality following hip fracture,^21^, ^22^ multivariable adjustment for educational level, number of children, county of residence, and whether the patient lived alone did not change our estimates substantially. Differences in physical activity level could probably be an explanatory factor, as patients in rural areas might be more physically active in daily life or traditionally have had more physically demanding occupations compared with urban residents. Physical activity is associated with lower mortality;^23^ and a better physical condition could also alleviate rehabilitation, which is important to improve the outcome after hip fracture.^24^

Lastly, it is possible that patients in small, rural municipalities have a different type of social network or higher degree of social interactions, supported by a study showing that elderly in rural areas reported more frequent social contact with their neighbors than their urban‐dwelling counterparts.^25^ This could result in better long‐term survival, as studies have demonstrated associations between a less amount of social contact and increased hip fracture mortality.^26^

### Strengths and limitations

The strengths of our study include the prospective design, the use of a nationwide validated hip fracture database, a large number of hip fracture patients followed up to 12.8 years, and nearly complete national datasets on sociodemographic factors. We recognize the need for comorbidity data, which could have added valuable knowledge when trying to explain the urban–rural mortality differences. Comorbidity data were unfortunately not available for the complete period 2002 to 2013 because of administrative reasons. Moreover, we do not have information on trauma mechanism, which is also a limitation in the study. Another possible limitation is a potentially long time span between the Population and Housing Census in 2001 and the occurrence of hip fracture, causing misclassification if the patient had moved during this period. However, elderly persons do not frequently move to other municipalities,^27^ and sensitivity analyses limited to hip fractures occurring during 2002 to 2006 showed the same patterns as the main analyses. Furthermore, the degree of urbanization is determined on a municipality level, not on an individual level. This is perhaps especially problematic in the semirural areas and may lead to misclassification of study participants, eg, in patients residing in mainly rural municipalities, but with a few, rather large, population centers. However, results from the sensitivity analysis (comparing strictly urban to strictly rural municipalities) support our main results.

In conclusion, time‐varying increased posthip fracture mortality in urban compared with rural municipalities was identified in both men and women in this large, register‐based cohort study. Mortality rates were higher in male compared with female hip fracture patients across all categories of urbanization. Further studies are required to explore whether the novel findings of an urban–rural difference in hip fracture mortality reflect disparities in health status or lifestyle, differences in posthip fracture health care or rehabilitation, or a combination of several factors. This knowledge could be useful when developing strategies to reduce the very high mortality after hip fracture.

## Disclosures

All authors state that they have no conflicts of interest.

## Authors' roles:

Acquisition of data: TKO, KH, AJS, HEM. Study design: TKO and SMS. Data preparation: TKO and SMS. Statistical analyses: SMS, HS, TKO. Drafting the manuscript: SMS and TKO. Data interpretation, critically revising manuscript content, approving final version of the manuscript: SMS, TKO, HEM, CD, HS, AJS, KH, JEM, GST, NE, SF, CGG, BS, PV. SMS takes responsibility for the integrity of the data analysis.
